# Roadmap for strengthening the vaccine supply chain in emerging countries: Manufacturers' perspectives

**DOI:** 10.1016/j.jvacx.2020.100068

**Published:** 2020-06-09

**Authors:** Stephen Jarrett, Lingjiang Yang, Sonia Pagliusi

**Affiliations:** a Gracious International Inc., 28 Jiafeng Road, Shanghai 200131, China; b ChengDu Institute of Biological Products Ltd., 379 3 Section, Jinhua Road, Jinjiang District, Chengdu 610023, China; c DCVMN International, Route de Crassier 7, 1262 Eysins-Nyon, Switzerland

**Keywords:** Supply chain, Stockpile, Traceability, Packaging technology, Environmental footprint

## Abstract

Manufacturers have a significant stake in the vaccine supply chain as their reputations rest on the effectiveness of their vaccines at the point of vaccination. The risks of low performing supply chains are detrimental for the safety and effectiveness of vaccines, with potential consequences to future supply in the case of adverse events. For this reason, a study was set up to explore the involvement of developing country manufacturers in the vaccine supply chain over the next decade to determine the areas where innovations could have a positive impact on the supply chain, focusing on the secondary stages of production where formulation, filling and packaging take place. An expert desk review identified eight areas of interest for the vaccine supply chain and informed the development of a survey to assess the relevance of the areas identified. The review also conjectured whether the overall effect of the identified areas is cost-neutral or resulting in net savings to countries. Overall, respondents identified five areas as of highest interest and subsequently an expert working group of representative manufacturers prioritized three of them. Specifically, traceability in the context of global digital health initiatives, stockpiling in the context of addressing vaccine shortages, stock-outs, outbreaks and epidemic prevention, and new packaging technologies are discussed in this report. It is important that vaccine manufacturers be actively engaged in global stakeholders' forums, as equal partners in determining the best ways for improving the vaccine supply chain.

## Highlights

• The study explored ways manufacturers could improve vaccine supply chain performance.
• It focused on the secondary stages of production: formulation, filling and packaging.
• 8 areas of interest for the vaccine supply chain were identified and 3 prioritized.
• Traceability, stockpiling and new packaging technologies are discussed.
• Specific proposals are made to engage in each of the 3 priority areas.

## 1 Introduction

In the supply of vaccines to developing countries, emerging country manufacturers are becoming increasingly important. Innovative vaccines have been developed, that are already pre-qualified by the World Health Organization (WHO), and newer vaccines are coming to market, expanding the global supply capacity.

Vaccine manufacturers from emerging countries, both publicly- and privately-owned, established a public health driven international network, the Developing Countries’ Vaccine Manufacturers Network (DCVMN) with a mission of protecting people from infections by supplying high-quality and affordable vaccines. DCVMN follows the United Nations classification of developing countries^1^, based on the Human Development Index and differing from the World Bank country income level classification. This report refers to emerging and/or developing countries interchangeably. One of the DCVMN’s strategic goals is to promote the stable and sustainable supply of high-quality vaccines to developing country populations, including ways to increase the efficiency and effectiveness of the vaccine supply chain. In 2018, DCVMN manufacturers provided over half the 2.4 billion doses procured by UNICEF^2^. In the case of the PAHO^3^ Revolving Fund, nearly 80% of the 275 million doses procured in 2018 were from DCVMN manufacturers.

While manufacturers' management of vaccine supply mostly stops at shipment or factory exit, except in the case of some national shipments, vaccines then start a long road to vaccination in country distribution chains which are fraught with challenges [1]. From 154 vaccine supply assessments in 89 countries conducted between 2009 and 2016, performance of nine indicators mostly fell below the recommended 80% threshold [2].

**Fig. 1:**
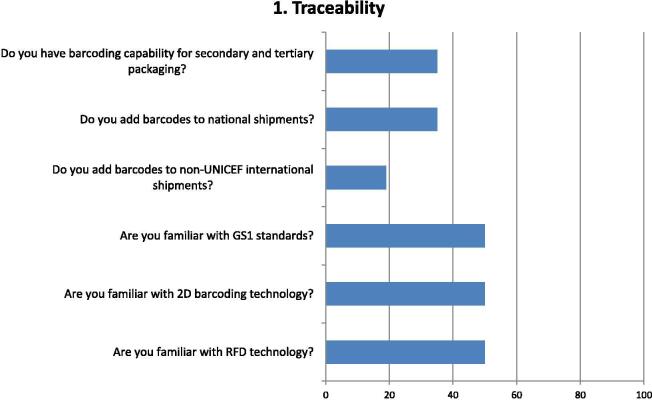
Questions and Responses of manufacturers related to traceability. Bar chart shows percentage of positive responses on the capability for product traceability. Six questions assessed factors considered on traceability, including barcoding capabilities for national and international supply, and actions, and familiarity with globally used standards and technologies supporting the tracking of vaccines through the supply chain: GS1 (www.gs1.org), 2 dimensional codes (2D) and radio frequency devices (RFD) put in shipments for tracking.

**Fig. 2:**
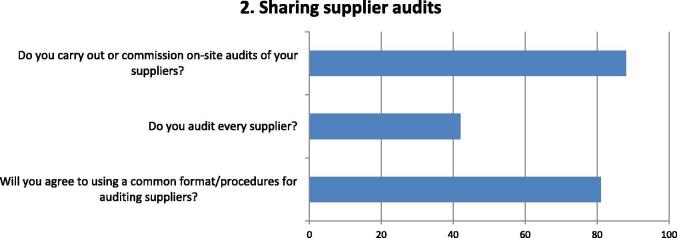
Questions and responses of manufacturers related to sharing supplier audits. Bar chart shows percentage of positive responses on the practices of sharing audit reports of manufacturers' suppliers (e.g. vials, ampoules, stoppers, packaging, etc.) Three major factors were considered related to the extent manufacturers carry out supplier audits and the level of agreement in having a common format/procedures for supplier audits.

**Fig. 3:**
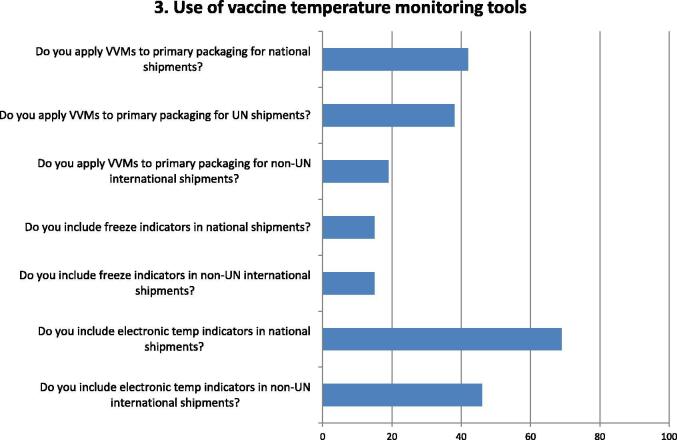
Questions and responses of manufacturers related to the use of temperature monitoring tools. Bar chart shows percentage of positive responses on the use of temperature monitoring tools. Seven questions assessed the use by manufacturers of temperature monitoring devices, at national or international supply of vaccines, particularly: Vaccine Vial Monitors (VVMs) on primary packaging, freeze indicators inserted into secondary packaging and electronic temperature indicators placed in shipping cartons.

**Fig. 4:**
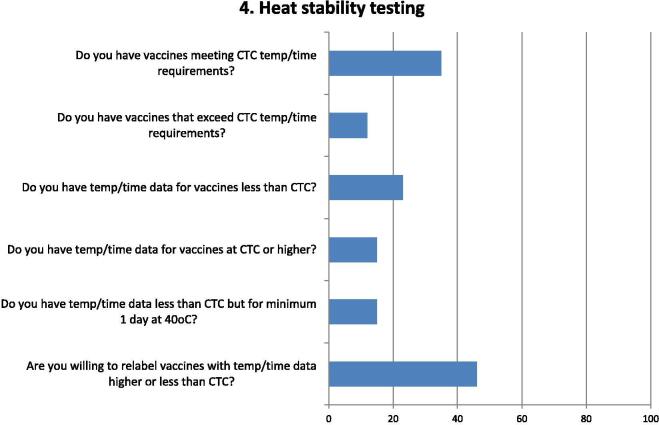
Questions and responses from manufacturers related to heat stability of vaccines complying with controlled temperature chain (CTC). Bar chart shows percentage of positive responses on heat stability testing of vaccines that comply with or exceed the WHO CTC requirements. Six questions assessed whether stability data are available and whether manufacturers would be willing to relabel their vaccines with additional heat stability data. The factors considered included the availability of temperature/time data on heat stability of vaccines, both in terms of CTC requirements (minimum of 3 days at 40 °C) as well as requirements that exceed or are below CTC requirements, in accordance with extended CTC (ECTC) stipulations. The second question on CTC is more specific than the first one, as it asks if manufacturers have the temperature/time data available. It is interesting to note from the survey that 9 manufacturers indicated having vaccines compliant with CTC requirements but only 4 indicated that they have the data.

**Fig. 5:**
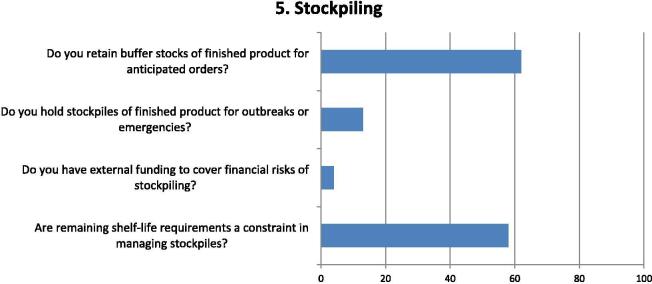
Questions and responses of manufacturers related to stockpiling of vaccines. Bar chart shows percentage of positive responses on stockpiling of vaccines either as static or rotating stocks. Four questions assessed the factors considered on stockpiling vaccines in finished product form, in anticipation of orders or to respond to outbreaks or emergencies; the degree to which external funding is available to cover stockpile costs and whether remaining shelf-life requirements from vaccine buyer causes a significant constraint to managing stockpiles.

**Fig. 6:**
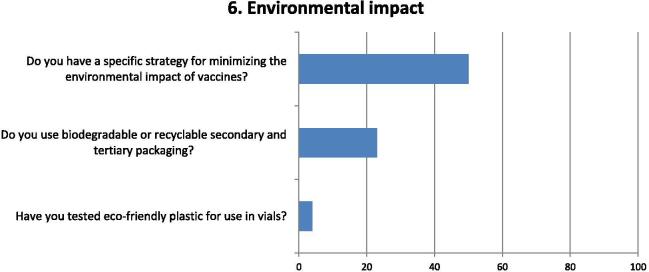
Questions and responses of manufacturers related to the environmental impact of vaccines disposal. Bar chart shows percentage of positive responses of manufacturers to questions relevant to lessening the environmental impact of vaccines. Three factors were considered on tackling the environmental impact of vaccines, both from a strategic perspective related to the existence of a specific strategy, as well as the use of recyclable or eco-friendly packaging materials and the use of environmental friendly primary (vials) packaging alternatives.

**Fig. 7:**
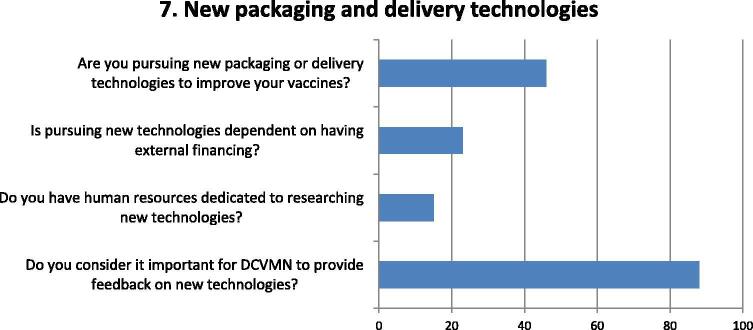
Questions and responses of manufacturers related to new packaging and delivery technologies. Bar chart shows percentage of positive responses of manufacturers to questions deemed relevant to their potential involvement in the development of new packaging and delivery technologies. Four question assessed factors considered on manufacturer involvement in pursuing the development of new packaging and delivery technologies, particularly regarding feedback on their feasibility and adoptability and cost, as well as the importance of access to external funding and capacity to follow developments.

**Fig. 8:**
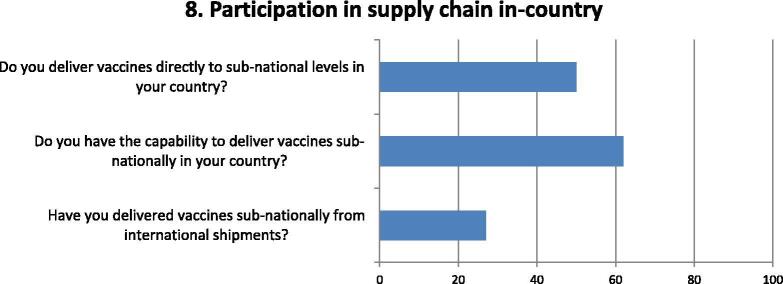
Questions and responses of manufacturers related to direct participation in the vaccine supply chain in countries. Bar chart shows the percentage of positive responses of manufacturers to questions deemed relevant to their respective capability and involvement in the vaccine supply chain. Three questions assessed factors considered whether manufacturers were already delivering vaccines to sub-national locations locally or internationally, or whether they have the capacity to deliver sub-nationally in the near future.

**Fig. 9:**
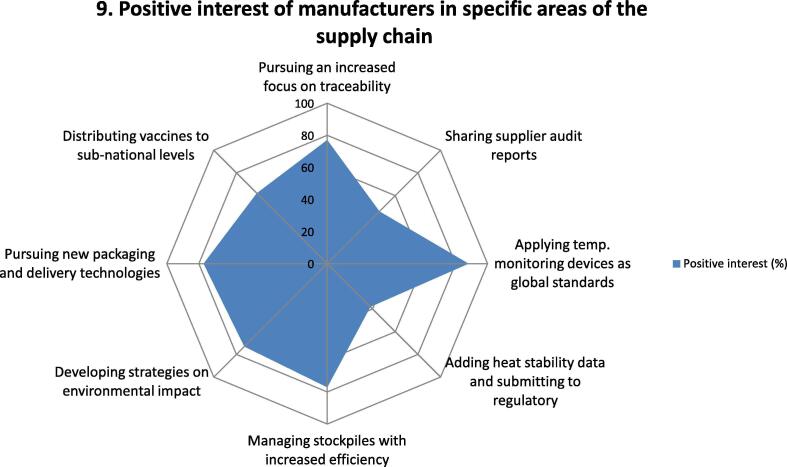
Questions and responses of manufacturers related to interest of respondents in DCVMN pursuing each of the supply chain areas assessed. Spider chart shows the percentage of positive responses of manufacturers to as to their interest in DCVMN focusing on each of the eight areas assessed above, deemed relevant to the vaccine supply chain. Each axe of the spider graph represents the level of interest in each of the areas: Traceability, sharing supplier audits, vaccine temperature monitoring tools, heat exposure or stability testing, Stockpiling, environmental impact of packaging, exploring new packaging and delivery technologies and direct participation in sub-national distribution. The spider chart depicts the percentage of positive responses corresponding to the degree of interest of manufacturers in pursuing each of the challenges identified, as shown in the blue-shaded area in the chart. This assessment facilitated the establishment of priorities for DCVMN to pursue and design the agenda of a future work plan. (For interpretation of the references to colour in this figure legend, the reader is referred to the web version of this article.)

The risks of low performing supply chains are detrimental for the safety and effectiveness of vaccines at time of vaccination, with potentially negative consequences to future supply and reputation. For this reason, DCVMN initiated a study to explore the involvement of members’ manufacturers in the vaccine supply chain over the next decade to determine the areas where their engagement could have a positive impact on the supply chain, focusing on the secondary stages of production where formulation, filling and packaging take place (See Fig. 1, Fig. 2, Fig. 3, Fig. 4, Fig. 5, Fig. 6, Fig. 7, Fig. 8, Fig. 9).

## 2 Methodology

Initially, an expert desk review identified eight areas relevant to the vaccine supply chain. This review informed the development of an anonymous survey to assess among DCVMN members the relevance of the areas identified. The survey was composed of 36 questions on manufacturers' general supply capabilities, actions and understanding of global standards. In addition, eight questions asked manufacturers' interest in pursuing each area through a vaccine supply chain expert group. Regulatory issues were not included as they are assessed separately by DCVMN [3].

Answers to the survey questions could only be YES or NO, to provide a quantitative snapshot of the areas identified. The anonymity of the survey meant that the profiles of respondents were unknown. The survey was circulated only in English, which may have limited responses from manufacturers without a reasonable command of the English language. It was open for 3 weeks from 7 to 28 March 2019, resulting in 26 respondents, a 60% response rate.

Following the survey, phone interviews, which delved deeper into the areas, were held by an independent consultant with nine manufacturers, of eighteen contacted, from Africa, Asia and the Americas, including six with vaccines pre-qualified by WHO, to seek feedback on qualitative aspects of the surveyed areas. Five representative manufacturers (from S.Africa, Brazil, India, China, Rep. Korea), constituting a working group, then met in Geneva, on 25th June 2019, and reviewed the results of the survey and subsequent interviews and determined the initial priorities proposed as the DCVMN members focus, taking into account initiatives being undertaken by the global immunization community.

Areas addressed by the survey and interviews

The desk review assessed the areas where manufacturer interventions could have a positive impact on the supply chain:

1. Traceability determines the ability to track vaccines through the supply chain from factory to point of vaccination including key information such as the Global Trade Item Number (GTIN), lot number and expiry date. With appropriate systems in place these data support inventory management, consumption history, wastage and demand forecasting, as well as being key for vaccine safety monitoring.
2. Sharing supplier audits can, in principle, reduce the cost burden for manufacturers and their suppliers in ensuring the continued quality of inputs they purchase for manufacturing, including equipment, vials, stoppers, and other supplies/accessories.
3. Vaccine temperature monitoring tools, including vaccine vial monitors (VVMs), freeze indicators and electronic temperature recorders, can detect product exposure to high temperatures or freezing during the transportation and storage of vaccines [4]. They are not, as yet, global standards, which is a concern especially for VVMs which indicate exposure to heat, given that cold chain failures can occur at anytime, anywhere, for a number of reasons and are not confined to developing/emerging countries.
4. Heat stability testing of all vaccines determines the shelf life under normal conditions of storage from + 2–8 °C, and for VVM specification at + 37 ^o^C. An additional determination for using vaccines out of the cold chain is an extended controlled temperature chain (ECTC) which can consider any temperature/time setting above the traditional + 2–8 °C cold chain that might facilitate vaccine distribution and where CTC is one specification [5]. Improving heat stability information of vaccines could potentially increase manufacturers' capacity to supply countries especially if ECTC provisions increase in demand.
5. Stockpiling of finished product, either through rotating buffer stocks or static stockpiles, can help to mitigate uncertain demand forecasts and stock-outs, which continue to affect many countries [6], but pose potential financial risk to manufacturers, if not used or do not comply with remaining shelf-life requirements. Stockpiling can also serve to respond to emergencies or outbreaks.
6. Environmental impact assesses the carbon footprint of vaccines and the potential for reducing waste. The increasing numbers of vaccines significantly add to the risk of increased waste and wastage in countries. Using biodegradable or recyclable secondary and tertiary packaging and insulation materials is one consideration, as are reusable shipping containers.
7. New packaging and delivery technologies are being developed to improve the vaccine supply chain and vaccination. There is increasing attention to new packaging technologies including blow-fill-seal (BFS) tube technology, multi-component containers and smart packaging, while intradermal delivery devices, microarray patches (MAPs) and sublingual delivery represent new delivery technologies [7]. BFS technology used for inactivated cholera vaccine has reduced package volume by 30% and shipping weight by over 50%, improving storage, transportation and waste management [8].
8. Direct participation in the vaccine supply chain may help countries address the constraints existing in vaccine supply chains through assessing the need for new structures and policies and applying system design techniques [9]. Shipments directly to sub-national levels, especially within large countries, can ease internal distribution for either routine shipments or for campaign needs.

## 3 Survey results and related comments

Predicted costs and savings for manufacturers were based on specific drivers for each of the eight areas (Table 1). In examining the eight areas described above, any innovations, that require new or additional equipment, increased maintenance, more complex packaging control procedures, may incur a cost. Stockpiling would add costs, because vaccine doses incur costs of filling and packaging without specific orders from customers. Savings relate to the ability to rapidly provide vaccines as needed by countries, avoiding sudden large shipments that would increase the costs of distribution and vaccination activities. This will necessarily conflict with pressures to reduce the cost of vaccines, with the risk that some manufacturers may exit from products if profits are further reduced [10].

**Table 1:** Perception of potential costs and savings:

This table shows the eight areas in the analysis (first column) assessed against the potential costs and savings across the secondary and tertiary stages of the supply chain (first row) with a view to suggesting where potential costs and savings related to the eight analyzed areas may occur. The costs and savings include the likely projected costs to innovation in the vaccine production stages that should trigger savings (green) at country level in storage, distribution and vaccination, as well as in following up AEFIs. Added costs to production (yellow) would raise vaccine prices which would run counter to the strong desire for affordable vaccines. These projections would have to be confirmed with evidence. Overall, innovations may mostly add costs to vaccine production in the formulation, filling, packaging and lot release stages, but may well result in savings in the distribution, storage, vaccination and post-marketing surveillance stages. These are projections and would have to be confirmed with evidence collected in countries. Stakeholders may reflect on cost trade-offs with innovation. Countries would need to be aware of these trade-offs.

If these additional costs to manufacturing hold up in reality, then vaccine prices could rise, but could be offset by increased efficiency and savings in other areas^4^. Evidence would have to be collected over time on this basic assumption, as to whether the overall effect is cost-neutral or actually resulting in net savings to countries.

The survey has been analyzed from the perspective of manufacturers' self-reported knowledge, capabilities and actions relevant to the eight areas identified, providing qualitative rather than quantitative data. Results are reported as a percentage of 'yes' answers to 36 questions, among the 26 respondents.

Half the respondents indicated familiarity with GS1 standards, 2D barcoding and radio frequency devices (RFD) technology (Fig. 1). Only 36%, however, indicated having barcoding capability, the same percentage adding barcodes to national shipments, while only a fifth used barcoding on shipments sent directly to international destinations.

Almost all respondents are engaged in auditing suppliers but less than half audit every supplier, rather focusing on those considered critical to their operations (Fig. 2). Quite a high degree of agreement (80%) was indicated to using common formats and procedures for auditing suppliers.

Respondents indicated that electronic temperature indicators placed in shipping cartons are the monitoring tool most widely used, both for national and international shipments (Fig. 3). VVMs are less used and dependent on buyers' demand, with UNICEF the principal international purchaser requiring VVMs on primary packaging (since 1997). Freeze indicators are less used in national and international shipments and depend on the vaccine being shipped and its sensitivity to freezing.

A third of respondents indicated that they had vaccines meeting the minimum temperature–time requirements of CTC, but only a third of those had vaccines that exceed CTC requirements (Fig. 4). The availability of temperature–time data, for vaccines outside the CTC specification – higher or lower, and which would be required for regulatory purposes was, however, not readily available. Almost half of the respondents did indicate a willingness to consider adding to labels the temperature–time data they have which could provide greater flexibility of vaccine use in countries.

Around 60% of respondents indicated that they keep finished products in stock in the anticipation of orders, but indicated current remaining shelf-life requirements is an on-going constraint to managing stockpiles effectively (Fig. 5). Almost a third of respondents indicated they hold stockpiles of finished products for outbreaks or emergencies but only one indicated the availability of external, or third-party, funding to mitigate the risks of holding stock.

While half of respondents indicated having a specific strategy for minimizing their environmental impact, only a quarter are using biodegradable or recyclable secondary and tertiary packaging, indicating that environmental concerns are more focused on country-level action (Fig. 6). Only one respondent indicated having looked at eco-friendly plastics for possible primary packaging.

Most respondents indicated it was important for DCVMN members to be consulted and provide feedback on new packaging and delivery technologies, especially with regard to cost and practicability (Fig. 7). Less than half of respondents though are pursuing new packaging and delivery technologies to improve their vaccines, some dependent on external funding and support as many lack the human resources dedicated to this pursuit.

Half of respondents indicated that they deliver vaccines to sub-national levels in their countries even though several more have the capability to do so (Fig. 8). This depends on the requirement and set-up of the supply chain in countries. Only a quarter reported delivering vaccines to sub -national locations for international shipments, following the instructions of purchasers.

An additional eight questions asked respondents their interest in DCVMN pursuing each of the eight areas (Fig. 9).

Overall, respondents' interest in pursuing the eight areas identified was highest in 5 of them with more than two-thirds indicating their interest in:

• Vaccine temperature exposure monitoring (85%);
• Traceability (77%);
• Stockpiling (77%);
• New packaging and delivery technologies (77%);
• Environmental impact (73%).

Direct participation in the supply chain was of interest to 62% of respondents, but less interest was indicated in sharing supplier audits (46%) and in adding heat stability data to labels or inserts (38%).

## 4 Validation of the online survey with a subset of respondents

Following the survey analysis, phone interviews took place with nine manufacturers, a 50% response rate, of 18 selected. The lower response rate compared to the anonymous survey may be due to some manufacturers preferring to remain anonymous in providing feedback or the higher investment in time. Interviews elicited detailed feedback on each area and determining which should be given priority by DCVMN. Five of the 9 interviewed manufacturers prioritized the areas. Despite the fact that only 5 manufacturers expressed their prioritization choices at the interviews, four of the five priorities coincided on traceability, vaccine exposure monitoring, stockpiling and new packaging and delivery technologies; environmental impact was therefore discarded as a priority, to narrow down the common priorities for the DCVMN. Noteworthy, the following main points were expressed in the interviews:

1. Stockpiling is a concern given both the cost of storage and the potential financial risk if stockpiled vaccines are not used or do not comply with the required remaining shelf life for shipment. Prepayment of stockpiles or at least a financial incentive to hold stock was specifically indicated by three manufacturers while all manufacturers stated the need for countries to accept lower requirements for remaining shelf life for stockpiled vaccines;
2. New packaging and delivery technologies had in the past not been perceived as a high priority for manufacturers due to the probable high costs involved; however they are now aware of the research being undertaken and all considered it important to actively evaluate such innovations relevant to vaccine supply to developing countries;
3. Effectively tracking vaccines through the supply chain (traceability) is important to all manufacturers especially related to AEFIs^5^ and pharmacovigilance monitoring, while noting that bar coding on primary packaging could be costly. Nevertheless, it was recognized by some that barcoding will increasingly be a requirement, including 2D matrix barcodes on primary packaging;
4. Vaccine temperature monitoring tools are used by manufacturers, mainly according to customer demand, such as VVMs on all UNICEF shipments but not on national shipments or other exports unless demanded. All manufacturers recognize that temperature monitoring tools provide significant security in distribution, which is a priority concern given low performance levels of many supply chains, but fell short of suggesting a greater standardization due to increased costs and lack of global demand;
5. Changes in heat stability labeling would require specific protocols from regulators (countries or WHO) and demand from countries. Two manufacturers indicated pursuing CTC for stable vaccines and one manufacturer noted that a wider range of heat stability data on each vaccine could be positive for marketing;
6. Environmental impact was seen by all manufacturers interviewed to be mainly in the hands of customers receiving vaccines, with secondary and tertiary carton packaging able to be recycled. One reference was made to the use of envirotainers which eliminate the use of plastic ice packs which are considered an environmental hazard;
7. Sharing supplier audits was indicated as potentially useful for reducing auditing costs but all manufactures pointed to significant challenges in establishing complex legal agreements between suppliers and manufacturers' quality assurance personnel across diverse jurisdictions, and to concerns around possible breaches of confidentiality;
8. Direct participation in the supply chain happens in the case of national shipments in producing countries with the potential for added services being provided by manufacturers. For international shipments, the customer essentially determines the point of disembarkation.

## 5 Final priority setting

A three-step prioritization process was followed: the survey, the interviews and the final selection of priorities. An expert working group of representative manufacturers reviewed the survey results and the subsequent in-depth interviews held with selected manufacturers, with the mandate to select 2–3 priority areas, where DCVMN can contribute globally. A key factor in the deliberations was related to feasibility and impact of each area, recognizing that opportunities for vaccine manufacturers are of higher impact when aligned with the global immunization community investments in specific initiatives. The four areas prioritized in both, the anonymous survey and the interviews, were then considered by the working group. The group deprioritized delivery technologies, as these need extensive R&D, thus not related to supply chain only, while maintaining new packaging technologies as priority. Temperature monitoring was considered a product specific development and requiring complex field studies, and engagement at this level was not feasible. Most pertinent areas were traceability in the context of global digital health initiatives, stockpiling in the context of addressing vaccine shortages, stock-outs, outbreaks and epidemics, and new packaging technologies. These three areas were, as a consequence, agreed as the initial priorities of the working group of DCVMN members, engaging actively in future analysis around the appropriateness, cost and adoptability of innovation related specifically to the functioning of the vaccine supply chain.

## 6 Vaccine supply roadmap considerations and discussion

The opportunities facing vaccine manufacturers in developing countries have to be considered against a significant degree of uncertainty, which can include weak demand forecasting, erratic ordering schedules, dependence on material and supplier relationships as well as the configuration and infrastructure of the supply chain. Added to this are externalities such as disease dynamics including outbreaks and epidemics, greater demand for data, environmental factors and tender procurement methods. Such uncertainties and externalities are confounded by a sustained under-investment in preparedness in countries for outbreak and epidemic response [11].

The ability and capacity to embrace opportunities remain a goal for many emerging vaccine manufacturers, but they often lack resources for significant changes to their operations [12]. While up-front investments (subsidies) are one response, this should be weighed against manufacturers' ability and capacity to adapt and adopt new technology [13]. Technology transfers constitute another option to support capacity building for manufacturers with scientific knowledge facilitating the transfer process [14].

There are two significant constraints. Firstly, emerging country vaccine manufacturers are diverse both in location across all developing regions, and in set-up with both privately-owned and state-owned companies, so seeking commonalities is not straightforward. Secondly, innovation and price pressure are competing goals and requires the understanding of international stakeholders that a balance is necessary for sustaining a healthy vaccine industry [15].

These constraints should be seen in a context where some manufacturers started supplying vaccines within the last decade and do not have significant experience in global supply chain.

DCVMN members’ feedback indicates their interest in pursuing ways to improve the vaccine supply chain, increasing their visibility and efficiency in the international immunization community by developing specific proposals and innovations pertinent to emerging countries.

1. Traceability

Traceability is a key feature in the implementation of immunization information systems, helping to plan and manage immunization activities and resources and ensuring that adequate quantities of vaccines are always available to meet demand [16]. It can streamline vaccine and ordering inventory, supply chain management, and safety monitoring, all directly of concern to manufacturers.

Health systems are increasingly integrating digital health which can lead to positive impact on immunization information systems, with immediate access to data on vaccine inventory, shortages and stock-outs. Gavi is already engaged in blockchain technology to track funds and vaccines. Within digital health there is a strong focus on mobile health (mHealth) using smartphones, which would enable health workers at all levels to track vaccines with mobile-phone applications, when packaging includes 2D barcoding; smartphone technology’s expanding sphere of influence could enable it to become the future of global health [17].

The World Health Assembly has broadly determined that the use of appropriate digital devices for public health can increase access to quality services, reduce maternal, child and neonatal mortality, increase health security and increase patient, family and community engagement [18]. Subsequently, WHO issued recommendations on digital interventions for health systems strengthening, including the use of stock notification and commodity management^6^.

While over 120 countries have reported having national digital health strategies [18], implementation concerns around digital health may be linked to the complexity of sustainability, including the need for leadership and governance, digital infrastructure, interoperability frameworks, partnerships and financing [19]. Digital health technologies are also potentially disruptive, due to new kinds of partnerships between organizations in the health, knowledge and telecommunications sectors [20].

So far, mHealth interventions have been largely on a pilot scale, many spearheaded by non-governmental bodies. The total market size of mobile health was estimated at US$23 billion by 2017, of which 42% was in the Asian Pacific, Latin American and African regions, with scaling up likely when governments embrace constructive policy for mobile health [21].

Barcoding significantly facilitates traceability and allows unit level data connection from manufacturer to end user, and is recommended by WHO on all vaccine packaging, except for primary packaging, following GS1 standards [22]. On vaccine tenders backed by Gavi financing and issued by UNICEF, it is required to have GS1^7^ standard barcoding on secondary packaging by latest 31 December 2021 [23].

There have been divergent views on barcodes on primary packaging (on the vaccine vial or ampoule); posing a technical challenge that could take several years to overcome, while a majority of manufacturers indicated implementation could occur much quicker. Some DCVMN member manufacturers are already pursuing barcoding on primary packaging to increase product security, given concerns about vaccines being diverted to parallel markets. This would also address secondary packaging being often discarded well before vaccination sessions, causing traceability to be lost before individuals are vaccinated. 2D matrix barcoding allows for fast and simple readability by downstream supply chain implementers and is already on primary packaging for vaccines used in developed countries [24].

The demands on manufacturers to support traceability down to the individual being vaccinated will become pressing as countries scale digital health, and it is an opportune time for manufacturers to:

• understand the specific demand of country immunization systems, including digital health systems, in terms of the traceability of vaccines down to individuals being vaccinated;

• model the options for including barcodes using GS1 standards at the primary packaging level;

• estimate the potential capital and operating costs involved;

• articulate financing options, which could include third-party grants to subsidize investment costs, advance market commitments to guarantee vaccine purchase volumes, the raising of vaccine costs, or potentially new financing innovations;

• showcase pilots in improving traceability.

2. Stockpiling

Stockpile investments are an integral part of comprehensive disease control strategies, providing countries with the capacity for rapid response to vaccine shortages or emergency situations [25]. Stockpiles address difficulties in purchasing vaccines at short notice, the need for very fast deployment and difficulties in foreseeing outbreaks ahead of time.

The shift in the global health landscape, with increased pressure from climate change, population increases and mass urbanization, heightens the risk of large-scale outbreaks and urban epidemics, continuously re-defining the role and size of vaccine stockpiles [26]. The WHO Blueprint R&D includes categories of priority diseases, of which 6 were in the midst of outbreaks at the same time in 2018 [27]. There are calls to consider stockpiles for all vaccines with elimination goals and help prevent and control endemic or epidemic diseases [28]. The focus of CEPI^8^ on outbreak response already signals the need for investigational stockpiles of candidate vaccines against the diseases it has targeted [29].

Gavi, which already supports cholera, yellow fever (YF) and meningococcal vaccine stockpiles, is working to mitigate the risk of outbreaks including creating the right market conditions to bolster vaccine stockpiles. Gavi provided an advance purchase commitment for an Ebola vaccine, ensuring its immediate availability and the eventual creation of a stockpile for future outbreaks. WHO has stated that the cholera stockpile has transformed a vicious cycle of low demand, low production, high price and inequitable distribution to a virtuous cycle of increased demand, increased production, reduced price and greater equity of access.^9^

UNICEF manages four stockpiles for outbreaks and humanitarian emergency situations – measles and measles & rubella (MR), oral cholera, YF and meningococcal vaccines - as well as a monovalent Oral Polio Vaccine (mOPV) stockpile for both bulk and finished product. The advantage of global stockpiles is common governance with an accountability framework based on good partnership to overcome a “first-come, first-served” approach [30].

Some stockpiles are static, not shipped until an outbreak or epidemic occurs. Rotating stockpiles are shipped for routine immunization programmes and specific volumes should be available within 72 h, as stipulated in UNICEF tenders [31]. The oral cholera and YF vaccine stockpile were specifically set up on a rotating stock basis. This differentiation between static and rotating stockpiles, based on programmatic use, is critical for epidemics of concern, including Ebola, Marburg, MERS^10^, SARS^11^, Zika, dengue, chikungunya, avian and pandemic influenza, cholera, measles, meningitis and YF. Whether stored as bulk and/or finished product depends on vaccine characteristics, time to fill and finish and the urgency for shipment. All stockpiles run the risk of product expiry, also loss if product does not meet countries' required remaining shelf-life conditions with additional costs of destruction.

While there is considerable investment in vaccines tied to elimination strategies and on outbreak response, short-term shortages and stock-outs of vaccines disrupting routine immunization activities are widespread. Between 2010 and 2015, countries in all regions of the world and of all economic levels experienced regular stock-outs of key vaccines [6]. This implies that many countries do not even hold sufficient buffer stock in the event of shortages or sudden increased demand. Effective stock management is one of the criteria for an effective vaccine supply chain [32]. Financing stockpiles is a critical factor as manufacturers consider stockpiles as product already purchased.

The likelihood of vaccine stockpiling becoming more prominent impels manufacturers to assess and determine their capabilities, conditions and best practices for retaining and expanding stockpiles, specifically to:

• collect more information on global and regional stockpiling policies for both creating and financing stockpiles;

• determine main contributions for manufacturers in creating, financing and maintaining stockpiles;

• identify potential efficiencies and financing options in stockpile management.

3. New packaging technologies

Gavi, WHO, Bill and Melinda Gates Foundation, PATH^12^, UNICEF and CHAI^13^ have formed an alliance creating a vaccine innovation prioritization strategy (VIPS) to drive vaccine product innovation to better meet country needs and support immunization coverage and equity goals [33]. The goal is to prioritize innovations in vaccine product attributes to provide greater clarity to manufacturers and partners to make investment decisions.

VIPS has prioritized 5 upstream and 4 downstream innovations, of 24 assessed, based on health impact, coverage and equity impact, safety impact, economic costs and potential breadth of innovation use, with cost issues of upmost importance from country perspectives [33]. Of these, MAPs, compact pre-filled auto-disable injection systems (cPADs), dual-chamber delivery devices, combined VVM and threshold indicator (TI) and barcodes are considered packaging innovations. Barcoding has been widely discussed in the context of traceability above.

MAPs may be a priority as a case has been made for the MR vaccine [34]. WHO has published a target product profile for MR-MAP. There are issues, however, that need resolution around patches containing vaccines especially related to clinical, regulatory, manufacturing and scale-up activities, as well as costs per unit when produced at large scale [35]. Manufacturers have expressed concerns around accountability in case of product failure – whether the patch mechanism or the vaccine is at fault.

The benefits of cPADs include delivering a correct dosage, low vaccine wastage, reduced logistics workload and reduced time by health workers to deliver vaccination. These benefits have been verified in Indonesia and Timor Leste where hepatitis B is delivered in a cPAD out-of-the-cold-chain in hard to reach areas [36]. In spite of the fact that both tetanus toxoid and hepatitis B vaccines in a cPAD have been prequalified by WHO, global demand has been very low because of the higher vaccine price, compounded by insufficient attention to overall costs. In late 2014, a pentavalent vaccine in a cPAD was pre-qualified by WHO [37], but the manufacturer stopped production of that presentation in 2017.

Dual-chamber delivery devices for lyophilized vaccines can simplify the reconstitution process. MR dual-chamber injection devices reduce open vial wastage at any volume and can lead to an increase in MR vaccine availability [38]. Of potential interest is the development by a research laboratory in India of a dual-chamber device for a heat-stable rotavirus vaccine that can stay out of the cold chain for 4 months at 45 °C [7].

While not an immediate priority of VIPS, BFS polymer containers have been shown to be the least expensive option for oral vaccines in terms of total cost of delivery and second to glass vials for injectable vaccines in multi-dose forms [39]. Given the advantage for oral vaccines, rotavirus and cholera manufacturers are already adopting BFS technology.

Improved packaging and presentation reduce stress on vaccine supply chains, through reduced volume packaging. There may be opportunities to reduce the packaging footprint in the cold chain. A smaller box for the safe delivery and storage of vaccines has been developed, halving the size of packaging, allowing twice as many doses to be shipped at once and occupying half the storage space, while removing all plastic to make it eco-friendly [40].

From surveys and interviews with DCVMN members during 2019, it was observed that many member manufacturers had not actively participated in the prioritization processes of VIPS, and would welcome to be engaged more actively in discussions around packaging innovations. This is particularly important as manufacturers will bear the costs of innovations and need to have the opportunity to voice the options they see on the practicalities, costs and financing of adopting innovations.

The development of new packaging technologies requires partnership between vaccine and technology manufacturers, with key considerations being programmatic suitability include cold chain volume, costs, IP rights and health impact [41]. More broadly, continued innovation in the vaccine industry can best be supported via a comprehensive and shared agenda across key stakeholders, with a view on demand clarity, economic incentives and early consultation on design [42]. Countries also need to have better opportunities to express their preferences and articulate demand for different products which they see as relevant in improving access and coverage rates [42].

Innovations in packaging are largely aimed at increasing the ease of vaccination by health care workers while reducing vaccine wastage and in some reducing storage volumes. DCVMN manufacturers have to be more active in partnering with innovation developers to ensure that innovations are feasible and cost-comparable with current technologies, specifically to:

• review the multiple innovations being developed by global stakeholders;

• become fully familiar with MAPs, cPADs, dual-chamber delivery devices, reduced packaging footprints and BFS;

• determine if additional innovations might be pursued by manufacturers;

• intervene in design, prototyping and piloting phases;

• identify any IP issues;

• estimate the projected capital and operating costs of selected innovations;

• signal potential financing options for the introduction of innovations.

## 7 Conclusion

Manufacturers have a significant stake in the vaccine supply chain as their reputations rest on the effectiveness of their vaccines at the point of vaccination. Manufacturers from developing countries are diverse in nature, being from distinct regions of the world and being either privately or state-owned. They agree, however, on finding ways to positively impact the vaccine supply chain through advances they can bring to vaccine production stages. This study provided an opportunity for manufacturers to express their views and served to identify areas where strengthening is warranted Traceability, stockpiling and the introduction of new packaging technologies have been selected as the initial priorities of a DCVMN vaccine supply chain expert group to engage manufacturers in a movement towards collective improvements.

In looking at the next decade, the overall context includes a global move to digital health which can provide significant opportunities for improving the traceability of vaccines. Here, the labeling of primary packaging to include 2D barcodes will likely become a requirement in the near future and manufacturers adopting this first may likely gain considerable market share. It is considered that major opportunities lie ahead for digital health innovators [43].

A second driver will be the increased demand for stockpiling vaccines, both as a measure to address shortages and stock-outs as well as for preparedness for outbreaks and epidemics. A third driver will be technological innovations that significantly improve the workload and time investment of health workers in vaccination and reduce vaccine wastage.

Key aspects for manufacturers to consider are the wider use of barcoding to enhance traceability, including the application to primary packaging, the increased use of stockpiling including buffer stocks, and new packaging technologies that include new primary containers. DCVMN manufacturers have to be more active in partnering with innovation developers to ensure that innovations are feasible and cost-comparable with current technologies. This implies to engage more actively in global stakeholders' forums, not as information recipients but as equal partners in determining the best ways forward for improving the vaccine supply chain.

## Declaration of Competing Interest

The authors declare that they have no known competing financial interests or personal relationships that could have appeared to influence the work reported in this paper.

## Footnotes

1 World Economic Situation and Prospect. United Nations; 2019. Available athttps://www.un.org/development/desa/dpad/wp-content/uploads/sites/45/WESP2019_BOOK-web.pdf.

2 United Nations Children’s Fund.

3 Pan American Health Organization.

4 The premise is that smart innovations in manufacturing can cause savings in the supply chain in immunization activities in countries.

6 https://www.who.int/reproductivehealth/publications/digital-interventions-health-system-strengthening/en/.

7 GS1 is a not-for-profit organization that develops and maintains global standards for business, www.gs1.org.

8 CEPI = Coalition for Epidemic Prevention Innovations.

9 https://www.who.int/publications/10-year-review/vaccines/en/index4.html.

10 MERS = Middle-East Respiratory Syndrome.

11 SARS = Severe Acute Respiratory Syndrome.

12 Formerly the Program for Appropriate Technology in Health.

13 CHAI = Clinton Health Access Initiative.

## Footnotes

5 AEFIs = Adverse events following immunization.

## References

[1] Rao R., Schreiber B., Lee B.Y. (2017). Immunization supply chains: why they matter and how they are changing, Editorial. Vaccine.

[2] WHO and UNICEF, Effective vaccine management (EVM): Global data analysis 2009-2016. WHO EVM database, November 2017. Cf. <https://www.who.int/immunization/programmes_systems/supply_chain/EVM-Global-Data-Analysis-2010-2018-EN.pdf?ua=1>.

[3] Dellepiane Nora, Pagliusi Sonia (2019). Opportunities for improving access to vaccines in emerging countries through efficient and aligned registration procedures: an industry perspective. Vaccine.

[4] WHO Guidelines on the international packaging and shipping of vaccines, WHO/IVB/05.23, 2005: <https://apps.who.int/iris/bitstream/handle/10665/69368/WHO_IVB_05.23_eng.pdf?sequence=1>.

[5] WHO Expert Committee on Biological Standardization, Guidelines on the stability evaluation of vaccines for use under extended controlled temperature conditions, Annex 5, Sixty-sixth report, WHO Technical Report Series No. 992, 2016: <https://www.who.int/biologicals/areas/vaccines/Annex_5_Guidelines_on_Stability_evaluation_vaccines_ECTC.pdf?ua=1>.

[6] Lydon P. (2017). Vaccine stock-outs around the world: are essential vaccines always available when needed?. Vaccine.

[7] Zehrung D. (2017). Exploring new packaging and delivery options for the immunization supply chain. Vaccine.

[8] Pagliusi S. (2018). Vaccines, inspiring innovation in health. Vaccine.

[9] WHO and UNICEF, System design approach to improve the immunization supply chain, Evidence Brief, WHO/IVB/18.01, 2018: <https://apps.who.int/iris/bitstream/handle/10665/272853/WHO-IVB-18.01-eng.pdf?ua=1>.

[10] Garrett L Inoculate against a global vaccine crisis. Foreign Policy, January 16; 2018. Cf. <https://www.lauriegarrett.com/other-writings/2018/5/2/inoculate-against-a-global-vaccine-crisis>.

[11] International Working Group on Financing Preparedness, From panic and neglect to investing in health security: financing pandemic preparedness at national level, World Bank, December; 2017: <http://pubdocs.worldbank.org/en/890291523304595565/FINAL-IWG-Report-3-5-18.pdf>.

[12] Stevenson MA. Geneva-Seattle collaboration in support of developing country manufacturing. Global Public Health, volume 13; 2018 – issue 4. DOI: 10.1080/17441692.2016.1245349. 10.1080/17441692.2016.1245349 27760489

[13] Luter N. (2017). An updated methodology to review developing country vaccine manufacturer viability. Vaccine.

[14] Pagliusi S. (2013). Developing Countries Vaccine Manufacturers Network: Doing good by making high-quality vaccine affordable for all. Vaccine.

[15] Saadatian-Elahi Mitra, Bloom David, Plotkin Stanley, Picot Valentina, Louis Jacques, Watson Michael (2017). Vaccination ecosystem health check: achieving impact today and sustainability for tomorrow. BMC Proc.

[16] PATH and WHO, Vision of future immunization supply and logistics systems: Action Plans, Project Optimize; September 2012: <https://path.azureedge.net/media/documents/TS_opt_action_plans.pdf>.

[17] Chung H, Mayes J, White A. How smartphone technology is changing health care in developing countries. J Global Health, Perspect, November 1; 2016. Available at <https://www.ghjournal.org/how-smartphone-technology-is-changing-healthcare-in-developing-countries/>.

[18] WHO, mHealth: Use of appropriate digital devices for public health, Report by the Director General, A71/20; 26 March 2018: <https://apps.who.int/iris/handle/10665/274134>.

[19] Broadband Commission for Sustainable Development Working Group on Digital Health, The promise of digital health: addressing non-communicable diseases to accelerate universal health coverage in LMICs, September 2018. Available at <https://broadbandcommission.org/Documents/publications/DigitalHealthReport2018.pdf>.

[20] Bloom G. (2019). Next steps towards universal health coverage call for global leadership. BMJ.

[21] GSMA and PwC, Touching lives through mobile health: Assessment of the global market opportunity; February 2012: <https://www.pwc.in/assets/pdfs/publications-2012/touching-lives-through-mobile-health-february-2012.pdf>.

[22] WHO, Assessing the programmatic suitability of vaccine candidates for WHO pre-qualification, Revision 2014, WHO/IVB/14.10; 2015: <https://apps.who.int/iris/bitstream/handle/10665/148168/WHO_IVB_14.10_eng.pdf?sequence=1>.

[23] Gavi, Vaccine manufacturer GS1 compliance, September 15, 2019, cf. <https://www.gavi.org/sites/default/files/document/supply-procurement/Gavi%20Announcement%20-%20Vaccine%20GS1%20Compliance.pdf>.

[24] Two-Dimentional (2D) Vaccine Barcodes. Global Standards Technical Implementation Guideline for global health commodities: product and location identification, labeling, and data exchange, version 2.1, March 2019, endorsed by Stop TB Partnership Global Drug Facility, The Global Fund, UNFPA, UNDP, USAID, PEPFAR and US CDC <https://www.cdc.gov/vaccines/programs/iis/2d-vaccine-barcodes/index.html>.

[25] Yen C. (2015). The development of global vaccine stockpiles. Lancet Infect Dis.

[26] Berkley S. (2017). Can vaccine stockpiles prevent the next pandemic?. World Economic Forum Annual Meeting.

[27] WHO, Research and Development Blueprint: 2018 annual review of diseases prioritized under the Research and Development Blueprint, Informal consultation, 6-7 February 2018, Geneva: <https://www.who.int/emergencies/diseases/2018prioritization-report.pdf>.

[28] Thompson K.M., Tebbens R.J.D. (2014). Framework for optimal global vaccine stockpile design for vaccine-preventable diseases: application to measles and cholera vaccines as contrasting examples. Risk Anal.

[29] Hatchett R., Lurie N. (2019). Outbreak response as an essential component of vaccine development. The Lancet.

[30] Nguyen T., Richardson S. (2019). Vaccine stockpile governance through partnership: the International Coordination Group on emergency vaccine provision and its impact. Int J Infect Diseases.

[31] Deehan H. Overview of vaccine production, UNICEF Supply Division; June 2017. Available at <https://www.who.int/influenza_vaccines_plan/objectives/SLPIVPP_Session5.6_Deehan.pdf>.

[32] Iwu C.J. (2019). Protocol for a systematic review of the effects of interventions for vaccine stock management. BMC.

[33] Menozzi-Arnaud M, Giersing B. VIPS – Vaccine Innovation Prioritization Strategy: focusing on vaccine product attributes, June 2019: <https://www.who.int/immunization/research/meetings_workshops/5_Menozzi_VIPS_PDVAC_2019.pdf?ua=1>.

[34] Giersing B.K. (2017). Challenges of vaccine presentation and delivery: how can we design vaccines to have optimal programmatic impact?. Vaccine.

[35] Peyraud N. (2019). Potential use of microarray patches for vaccine delivery in low- and middle-income countries. Vaccine.

[36] Childs L., Roesel S., Tohme R.A. (2018). Status and progress of hepatitis B control through vaccination in the South-East Asia Region, 1992–2015. Vaccine.

[37] World Health Organization. DtwP-HepB-Hib vaccine available in a compact, pre-filled, auto-disable injection technology (cPAD), Information Bulletin, March 2015: <https://www.who.int/immunization/programmes_systems/service_delivery/InfoBulletin_Uniject_March2015_FINAL_ENG.pdf?ua=1>.

[38] Wedlock P.T. (2018). Dual-chamber injection device for measles-rubella vaccine: The potential impact of introducing varying sizes of the devices in 3 countries. Vaccine.

[39] Sedita J. (2018). Cost of goods sold and total cost of delivery for oral and parenteral vaccine packaging formats. Vaccine.

[40] Bigger L. (2018). Potential impact of new technologies on delivering #immunization. IFPMA.

[41] Azimi T et al. Refueling the innovation engine in vaccines, McKinsey & Company; May 2019 cf. <https://www.mckinsey.com/industries/pharmaceuticals-and-medical-products/our-insights/refueling-the-innovation-engine-in-vaccines#>.

[42] Giersing B. Novel initiatives to improve vaccine coverage and equity, DCVMN meeting, Kunming, China, 29 October – 1 November 2018: <https://www.dcvmn.org/IMG/pdf/10_giersing_dcvmn_tse_vips.pdf>.

[43] Rahimi K. (2019). Digital health and the elusive quest for cost savings. The Lancet Digital Health.

